# (1*Z*)-1-(1-Benzo­furan-2-yl)ethanone oxime

**DOI:** 10.1107/S1600536813033102

**Published:** 2013-12-14

**Authors:** D. B. Arunakumar, R. Desai Nivedita, S. Sreenivasa, S. Madan Kumar, N. K. Lokanath, P. A. Suchetan

**Affiliations:** a Department of Studies and Research in Chemistry, Tumkur University, Tumkur, Karnataka 572 103, India; b Department of Studies in Physics, University of Mysore, Manasagangotri, Mysore, India; c Department of Studies and Research in Chemistry, U.C.S, Tumkur University, Tumkur, Karnataka 572 103, India

## Abstract

The title compound, C_10_H_9_NO_2_, is almost planar (r.m.s. deviation for the non-H atoms = 0.027 Å) and the conformation across the C=N bond is *syn*. Further, the O atom of the benzo­furan ring is *syn* to the CH_3_ group in the side chain. In the crystal, mol­ecules are linked into *C*(3) chains propagating in [010] by O—H⋯N hydrogen bonds.

## Related literature   

For the broad range of biological activities of the benzo­furan moiety, see: Mehnaz *et al.* (2011 ▶). For the anti­fungal activity of (benzo­furan-2-yl) keoximes, see: Demirayak *et al.* (2002 ▶).
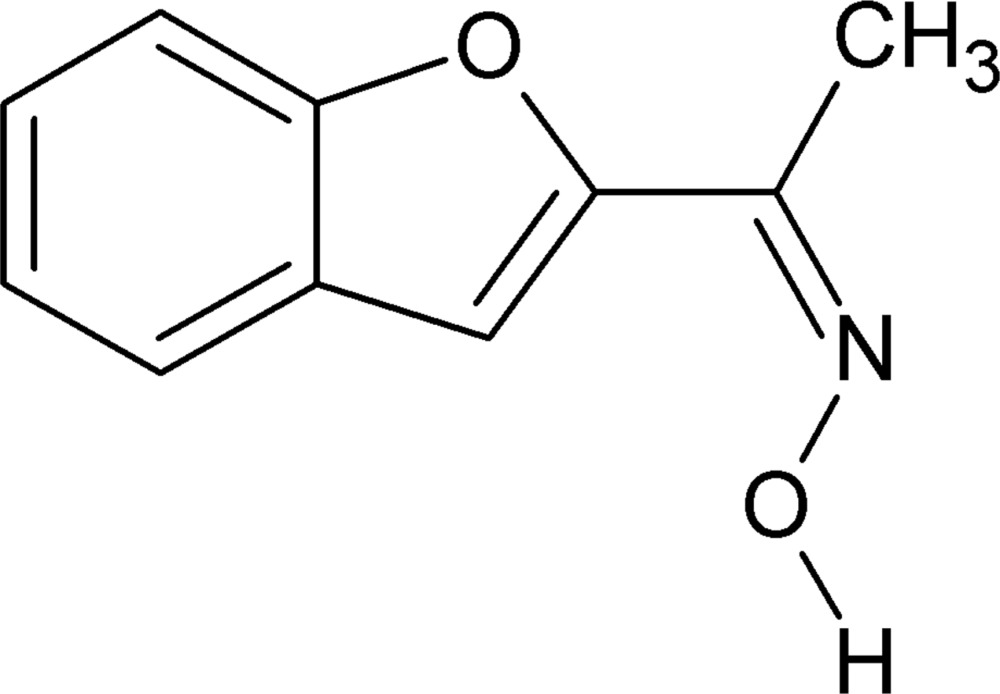


## Experimental   

### 

#### Crystal data  

C_10_H_9_NO_2_
*M* *_r_* = 175.18
Monoclinic, 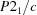

*a* = 9.5727 (12) Å
*b* = 4.7303 (8) Å
*c* = 18.756 (2) Å
β = 96.178 (6)°
*V* = 844.4 (2) Å^3^
*Z* = 4
Cu *K*α radiation
μ = 0.80 mm^−1^
*T* = 293 K
0.35 × 0.27 × 0.22 mm

#### Data collection  

Bruker APEXII CCD diffractometer
Absorption correction: multi-scan (*SADABS*; Bruker, 2009 ▶) *T*_min_ = 0.772, *T*_max_ = 0.839
2478 measured reflections
1333 independent reflections
1199 reflections with *I* > 2σ(*I*)
*R*_int_ = 0.019

#### Refinement  

*R*[*F* ^2^ > 2σ(*F* ^2^)] = 0.062
*wR*(*F* ^2^) = 0.146
*S* = 1.07
1333 reflections
120 parameters
H-atom parameters constrained
Δρ_max_ = 0.22 e Å^−3^
Δρ_min_ = −0.40 e Å^−3^

### 

Data collection: *APEX2* (Bruker, 2009 ▶); cell refinement: *APEX2* and *SAINT-Plus* (Bruker, 2009 ▶); data reduction: *SAINT-Plus* and *XPREP* (Bruker, 2009 ▶); program(s) used to solve structure: *SHELXS97* (Sheldrick, 2008 ▶); program(s) used to refine structure: *SHELXL97* (Sheldrick, 2008 ▶); molecular graphics: *Mercury* (Macrae *et al.*, 2008 ▶); software used to prepare material for publication: *SHELXL97*.

## Supplementary Material

Crystal structure: contains datablock(s) I, New_Global_Publ_Block. DOI: 10.1107/S1600536813033102/hb7169sup1.cif

Structure factors: contains datablock(s) I. DOI: 10.1107/S1600536813033102/hb7169Isup2.hkl

Supporting information file. DOI: 10.1107/S1600536813033102/hb7169Isup3.cml

Additional supporting information:  crystallographic information; 3D view; checkCIF report

## Figures and Tables

**Table 1:** Hydrogen-bond geometry (Å, °)

*D*—H⋯*A*	*D*—H	H⋯*A*	*D*⋯*A*	*D*—H⋯*A*
O2—H2⋯N1^i^	0.82	2.03	2.838 (2)	166

Symmetry code: (i) 
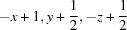
.

**Fig. 1.**
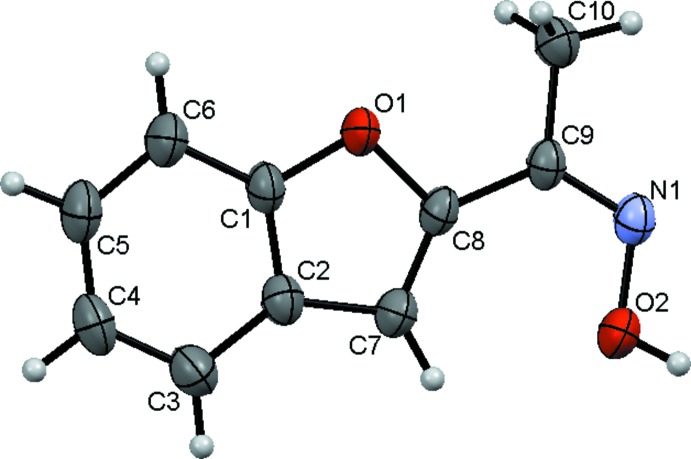
Molecular structure of the title compound, showing displacement ellipsoids drawn at the 50% probability level.

**Fig. 2.**
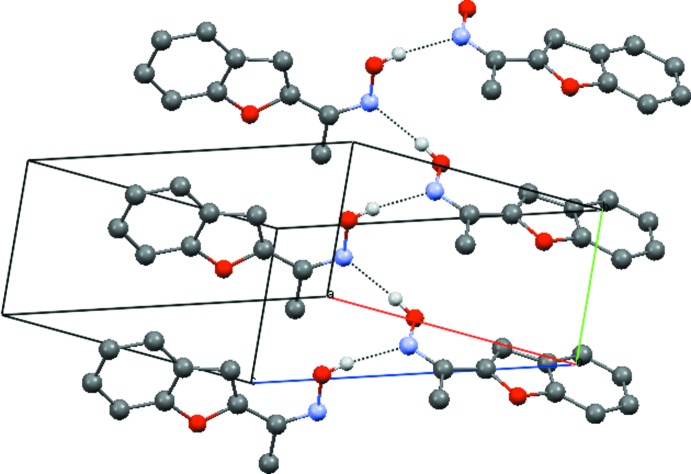
Linking of molecules into C(3) chains through O—H···N hydrogen bonds. H-atoms not involved in H-bonding are omitted for clarity purpose.

## supplementary crystallographic information

### 1. Comment

Benzofurans are bicyclic ring systems with multiple applications. The literature indicates that compounds having benzofuran nucleus possesses broad range of biological activities like anifungal, antiarrythmic, uricisuric, vasodilator and antimigraine agent (Mehnaz *et al.*, 2011). Further, (benzofuran-2-yl) keoximes shows good antifungal activity (Demirayak *et al.*, 2002). Keeping this thing in mind, the title compound was synthesized and its crystal structure determined.

In the title compound, C_10_H_9_NO_2_, the molecule is almost planar (r.m.s. deviation for the non-H atoms = 0.027 Å) and the conformation across the C=N bond is *syn*. Further, the oxygen atom of the benzofuran ring is *syn* to the CH_3_ group in the side chain. In the crystal structure, the molecules are linked into C(3) chains through O2—H2···N1 hydrogen bonds.

### 2. Experimental

2-Acetylbenzofuran (0.0062 mmol), hydroxyaminehydrochloride (0.0093 mmol) and anhydrous potassium carbonate (0.0093 mmol) were taken in a round bottom flask containing ethanol and water taken in the ratio 3:1. The reaction mixture was refluxed for 3 hrs·The progress of the reaction was monitored by thin layer chromatography. The reaction mixture was poured into ice cold water. The title compound separated as white solid. It was filtered, washed with water, dried and recrystallized from ethanol.

Colourless prisms were obtained from the solvent system: ethyl acetate: methanol (4:1) by recrystallisation.

### 3. Refinement

The H atoms were positioned with idealized geometry using a riding model with C—H = 0.93–0.96 Å and O—H = 0.82 Å. The isotropic displacement parameters for all H atoms were set to 1.2 times *U*_eq_ (C_aromatic_) and 1.5 times *U*_eq_ (C_methyl_, O)..

### Crystal data

**Table:**

C_10_H_9_NO_2_	Prism
*M**_r_* = 175.18	*D*_x_ = 1.378 Mg m^−^^3^
Monoclinic, *P*2_1_/*c*	Melting point: 473 K
Hall symbol: -P 2ybc	Cu *K*α radiation, λ = 1.54178 Å
*a* = 9.5727 (12) Å	Cell parameters from 1331 reflections
*b* = 4.7303 (8) Å	θ = 4.7–64.6°
*c* = 18.756 (2) Å	µ = 0.80 mm^−^^1^
β = 96.178 (6)°	*T* = 293 K
*V* = 844.4 (2) Å^3^	Prism, colourless
*Z* = 4	0.35 × 0.27 × 0.22 mm
*F*(000) = 368	

### Data collection

**Table:**

Bruker APEXII CCD diffractometer	1333 independent reflections
Radiation source: fine-focus sealed tube	1199 reflections with *I* > 2σ(*I*)
Graphite monochromator	*R*_int_ = 0.019
φ and ω scans	θ_max_ = 64.6°, θ_min_ = 4.7°
Absorption correction: multi-scan (*SADABS*; Bruker, 2009)	*h* = −11→10
*T*_min_ = 0.772, *T*_max_ = 0.839	*k* = −5→2
2478 measured reflections	*l* = −20→21

### Refinement

**Table:**

Refinement on *F*^2^	Primary atom site location: structure-invariant direct methods
Least-squares matrix: full	Secondary atom site location: difference Fourier map
*R*[*F*^2^ > 2σ(*F*^2^)] = 0.062	Hydrogen site location: inferred from neighbouring sites
*wR*(*F*^2^) = 0.146	H-atom parameters constrained
*S* = 1.07	*w* = 1/[σ^2^(*F*_o_^2^) + (0.1028*P*)^2^ + 0.2023*P*] where *P* = (*F*_o_^2^ + 2*F*_c_^2^)/3
1333 reflections	(Δ/σ)_max_ < 0.001
120 parameters	Δρ_max_ = 0.22 e Å^−^^3^
0 restraints	Δρ_min_ = −0.40 e Å^−^^3^

### Special details

**Table:**

Geometry. All s.u.'s (except the s.u. in the dihedral angle between two l.s. planes) are estimated using the full covariance matrix. The cell s.u.'s are taken into account individually in the estimation of s.u.'s in distances, angles and torsion angles; correlations between s.u.'s in cell parameters are only used when they are defined by crystal symmetry. An approximate (isotropic) treatment of cell s.u.'s is used for estimating s.u.'s involving l.s. planes.
Refinement. Refinement of *F*^2^ against ALL reflections. The weighted *R*-factor *wR* and goodness of fit *S* are based on *F*^2^, conventional *R*-factors *R* are based on *F*, with *F* set to zero for negative *F*^2^. The threshold expression of *F*^2^ > 2σ(*F*^2^) is used only for calculating *R*-factors(gt) *etc*. and is not relevant to the choice of reflections for refinement. *R*-factors based on *F*^2^ are statistically about twice as large as those based on *F*, and *R*- factors based on ALL data will be even larger.

### Fractional atomic coordinates and isotropic or equivalent isotropic displacement parameters (Å^2^)

**Table:**

	*x*	*y*	*z*	*U*_iso_*/*U*_eq_	
C1	1.04643 (18)	0.3538 (4)	0.41182 (10)	0.0348 (5)	
C2	1.06183 (18)	0.5553 (4)	0.35964 (10)	0.0354 (5)	
C3	1.1963 (2)	0.6670 (5)	0.35443 (12)	0.0458 (6)	
H3	1.2109	0.8023	0.3200	0.055*	
C4	1.3051 (2)	0.5706 (5)	0.40151 (12)	0.0464 (6)	
H4	1.3950	0.6408	0.3985	0.056*	
C5	1.2848 (2)	0.3703 (5)	0.45370 (12)	0.0473 (6)	
H5	1.3612	0.3125	0.4851	0.057*	
C6	1.1547 (2)	0.2556 (5)	0.46007 (12)	0.0464 (6)	
H6	1.1406	0.1206	0.4946	0.056*	
C7	0.92452 (19)	0.5946 (4)	0.32240 (10)	0.0377 (5)	
H7	0.9003	0.7176	0.2844	0.045*	
C8	0.83702 (18)	0.4191 (4)	0.35294 (9)	0.0318 (5)	
C9	0.68805 (17)	0.3508 (4)	0.34237 (9)	0.0312 (5)	
C10	0.63408 (19)	0.1283 (5)	0.38897 (11)	0.0402 (5)	
H10A	0.5356	0.0996	0.3752	0.060*	
H10B	0.6482	0.1879	0.4381	0.060*	
H10C	0.6838	−0.0453	0.3835	0.060*	
N1	0.59615 (15)	0.4690 (3)	0.29671 (8)	0.0351 (4)	
O1	0.90954 (12)	0.2666 (3)	0.40845 (7)	0.0375 (4)	
O2	0.65313 (13)	0.6771 (3)	0.25538 (7)	0.0417 (4)	
H2	0.5897	0.7764	0.2361	0.063*	

### Atomic displacement parameters (Å^2^)

**Table:**

	*U*^11^	*U*^22^	*U*^33^	*U*^12^	*U*^13^	*U*^23^
C1	0.0229 (9)	0.0405 (11)	0.0394 (10)	−0.0014 (7)	−0.0031 (7)	−0.0013 (8)
C2	0.0281 (9)	0.0408 (11)	0.0362 (9)	−0.0008 (7)	−0.0014 (7)	−0.0015 (8)
C3	0.0338 (10)	0.0546 (14)	0.0491 (12)	−0.0073 (9)	0.0052 (8)	0.0056 (10)
C4	0.0256 (9)	0.0568 (14)	0.0560 (12)	−0.0056 (8)	0.0009 (8)	−0.0058 (10)
C5	0.0294 (10)	0.0572 (14)	0.0526 (12)	0.0018 (9)	−0.0084 (8)	−0.0023 (10)
C6	0.0316 (11)	0.0553 (14)	0.0497 (12)	−0.0009 (9)	−0.0075 (8)	0.0113 (10)
C7	0.0312 (10)	0.0441 (11)	0.0361 (9)	−0.0003 (8)	−0.0036 (7)	0.0064 (8)
C8	0.0275 (9)	0.0341 (10)	0.0318 (9)	0.0022 (7)	−0.0059 (7)	−0.0009 (7)
C9	0.0255 (9)	0.0332 (10)	0.0332 (9)	0.0006 (7)	−0.0041 (7)	−0.0045 (7)
C10	0.0317 (10)	0.0418 (12)	0.0455 (11)	−0.0051 (8)	−0.0034 (8)	0.0028 (8)
N1	0.0286 (8)	0.0373 (10)	0.0376 (8)	−0.0004 (6)	−0.0043 (6)	−0.0024 (6)
O1	0.0254 (7)	0.0431 (9)	0.0417 (8)	−0.0027 (5)	−0.0068 (5)	0.0092 (6)
O2	0.0326 (7)	0.0471 (9)	0.0432 (8)	0.0027 (6)	−0.0059 (6)	0.0107 (6)

### Geometric parameters (Å, º)

**Table:**

C1—O1	1.369 (2)	C7—C8	1.350 (3)
C1—C6	1.381 (3)	C7—H7	0.9300
C1—C2	1.385 (3)	C8—O1	1.390 (2)
C2—C3	1.404 (3)	C8—C9	1.455 (2)
C2—C7	1.433 (3)	C9—N1	1.288 (2)
C3—C4	1.370 (3)	C9—C10	1.495 (3)
C3—H3	0.9300	C10—H10A	0.9600
C4—C5	1.391 (3)	C10—H10B	0.9600
C4—H4	0.9300	C10—H10C	0.9600
C5—C6	1.375 (3)	N1—O2	1.400 (2)
C5—H5	0.9300	O2—H2	0.8200
C6—H6	0.9300		
			
O1—C1—C6	125.18 (18)	C8—C7—C2	106.98 (17)
O1—C1—C2	110.42 (15)	C8—C7—H7	126.5
C6—C1—C2	124.40 (17)	C2—C7—H7	126.5
C1—C2—C3	118.42 (17)	C7—C8—O1	110.76 (15)
C1—C2—C7	105.77 (16)	C7—C8—C9	136.28 (17)
C3—C2—C7	135.81 (19)	O1—C8—C9	112.96 (15)
C4—C3—C2	118.0 (2)	N1—C9—C8	125.71 (18)
C4—C3—H3	121.0	N1—C9—C10	116.12 (15)
C2—C3—H3	121.0	C8—C9—C10	118.16 (15)
C3—C4—C5	121.75 (18)	C9—C10—H10A	109.5
C3—C4—H4	119.1	C9—C10—H10B	109.5
C5—C4—H4	119.1	H10A—C10—H10B	109.5
C6—C5—C4	121.77 (19)	C9—C10—H10C	109.5
C6—C5—H5	119.1	H10A—C10—H10C	109.5
C4—C5—H5	119.1	H10B—C10—H10C	109.5
C5—C6—C1	115.7 (2)	C9—N1—O2	113.26 (14)
C5—C6—H6	122.2	C1—O1—C8	106.06 (14)
C1—C6—H6	122.2	N1—O2—H2	109.5
			
O1—C1—C2—C3	−179.18 (17)	C2—C7—C8—O1	0.1 (2)
C6—C1—C2—C3	0.6 (3)	C2—C7—C8—C9	−179.9 (2)
O1—C1—C2—C7	0.5 (2)	C7—C8—C9—N1	−3.0 (4)
C6—C1—C2—C7	−179.7 (2)	O1—C8—C9—N1	177.02 (16)
C1—C2—C3—C4	−0.1 (3)	C7—C8—C9—C10	178.2 (2)
C7—C2—C3—C4	−179.7 (2)	O1—C8—C9—C10	−1.7 (2)
C2—C3—C4—C5	−0.6 (3)	C8—C9—N1—O2	0.6 (3)
C3—C4—C5—C6	1.0 (4)	C10—C9—N1—O2	179.33 (15)
C4—C5—C6—C1	−0.5 (3)	C6—C1—O1—C8	179.8 (2)
O1—C1—C6—C5	179.47 (19)	C2—C1—O1—C8	−0.4 (2)
C2—C1—C6—C5	−0.3 (3)	C7—C8—O1—C1	0.2 (2)
C1—C2—C7—C8	−0.3 (2)	C9—C8—O1—C1	−179.84 (14)
C3—C2—C7—C8	179.2 (2)		

### Hydrogen-bond geometry (Å, º)

**Table:**

*D*—H···*A*	*D*—H	H···*A*	*D*···*A*	*D*—H···*A*
O2—H2···N1^i^	0.82	2.03	2.838 (2)	166

Symmetry code: (i) −*x*+1, *y*+1/2, −*z*+1/2.
